# Towards performing high-resolution inelastic X-ray scattering measurements at hard X-ray free-electron lasers coupled with energetic laser drivers

**DOI:** 10.1107/S1600577522004453

**Published:** 2022-05-18

**Authors:** A. Descamps, B. K. Ofori-Okai, J. K. Baldwin, Z. Chen, L. B. Fletcher, S. H. Glenzer, N. J. Hartley, J. B. Hasting, D. Khaghani, M. Mo, B. Nagler, V. Recoules, R. Redmer, M. Schörner, P. Sun, Y. Q. Wang, T. G. White, E. E. McBride

**Affiliations:** a SLAC National Accelerator Laboratory, 2575 Sand Hill Road, Menlo Park, CA 94025, USA; b Aeronautics and Astronautics Department, Stanford University, 450 Serra Mall, Stanford, CA 94305, USA; c Los Alamos National Laboratory, Bikini Atoll Road, Los Alamos, NM 87545, USA; d CEA/DAM DIF, F-91297 Arpajon Cedex, France; e Université Paris-Saclay, CEA, Laboratoire Matière en Conditions Extrêmes, 91680 Bruyères-le-Châtel, France; f Institut für Physik, Universität Rostock, Albert-Einstein-Straße 23, 18059 Rostock, Germany; g University of Nevada, Reno, NV 89557, USA; h PULSE Institute, SLAC National Accelerator Laboratory, 2575 Sand Hill Road, Menlo Park, CA 94025, USA

**Keywords:** XFEL, high-resolution inelastic X-ray scattering, extreme conditions, thermal diffuse scattering

## Abstract

High-resolution inelastic X-ray scattering is an established technique in the synchrotron community, used to investigate collective low-frequency responses of materials. When fielded at hard X-ray free-electron lasers (XFELs) and combined with high-intensity laser drivers, it becomes a promising technique for investigating matter at high temperatures and high pressures. This technique gives access to important thermodynamic properties of matter at extreme conditions, such as temperature, material sound speed, and viscosity. The successful realization of this method requires the acquisition of many identical laser-pump/X-ray-probe shots, allowing the collection of a sufficient number of photons necessary to perform quantitative analyses. Here, a 2.5-fold improvement in the energy resolution of the instrument relative to previous works at the Matter in Extreme Conditions (MEC) endstation, Linac Coherent Light Source (LCLS), and the High Energy Density (HED) instrument, European XFEL, is presented. Some aspects of the experimental design that are essential for improving the number of photons detected in each X-ray shot, making such measurements feasible, are discussed. A careful choice of the energy resolution, the X-ray beam mode provided by the XFEL, and the position of the analysers used in such experiments can provide a more than ten-fold improvement in the photometrics. The discussion is supported by experimental data on 10 µm-thick iron and 50 nm-thick gold samples collected at the MEC endstation at the LCLS, and by complementary ray-tracing simulations coupled with thermal diffuse scattering calculations.

## Abstract 2

High-resolution inelastic X-ray scattering measurements at hard X-ray free-electron lasers coupled with energetic laser drivers have shown a 2.5-fold improved energy resolution compared with previous experiments at similar XFEL instruments. Aspects of the experimental design that can be adjusted to improve the number of recorded photons on the detector are discussed.

## 1. Introduction

With the advent of X-ray free-electron lasers (XFELs) and their unique capability to deliver high-brightness X-ray pulses, techniques developed at synchrotron light sources have been adapted to take advantage of the tens of femtoseconds temporal resolution, allowing the investigation of ultrafast phenomena. Among such techniques, inelastic X-ray scattering with milli-electronvolt (meV) resolution has been extensively used at synchrotron light sources to investigate the behavior of liquids (Monaco *et al.*, 1999 ▸; Ruocco *et al.*, 2003 ▸) as well as matter at high pressures using diamond anvil cells (Ohtani *et al.*, 2015 ▸; Shen & Mao, 2017 ▸). Recently, this technique, coupled with XFELs, has also been proposed as a model-independent temperature diagnostic in transient dense systems generated in extreme conditions experiments (Descamps *et al.*, 2020 ▸). In contrast to synchrotron facilities, where the data acquisition rate is limited by the repetition rate of the storage ring (typically a few MHz) (Bizek, 1996 ▸), the data acquisition rate in experiments coupling energetic lasers, used to generate matter at extremes of pressure and temperature, with XFELs is limited by the repetition rate of the laser drivers (typically a few Hz, or less) (Glenzer *et al.*, 2016 ▸) and by the target delivery. This reduction in the repetition rate has a profound impact on the design of the experimental setup as the expected number of photons on the detector per X-ray shot is comparatively low. To date, an energy resolution on the order of ∼60 meV using the (533) reflection of silicon at ∼7.5 keV has been demonstrated at XFEL facilities (McBride *et al.*, 2018 ▸; Wollenweber *et al.*, 2021 ▸), and used to measure phonon modes in diamond. However, this energy resolution limits the measurements to materials with large sound speeds, *i.e.* diamond. For most materials, with lower phonon energies, an improved energy resolution is essential for extracting quantitative information from inelastic spectra. In this work, we present an improved energy resolution, by using higher-energy X-ray photons. A few considerations that would maximize the number of photons on the detector at XFELs are also presented in the last two sections. The discussion is based on high-resolution inelastic X-ray scattering measurements of phonon modes in iron and gold at ambient conditions performed at the Matter in Extreme Conditions (MEC) endstation at the Linac Coherent Light Source (LCLS).

## 2. Experimental setup

**Figure 1:**
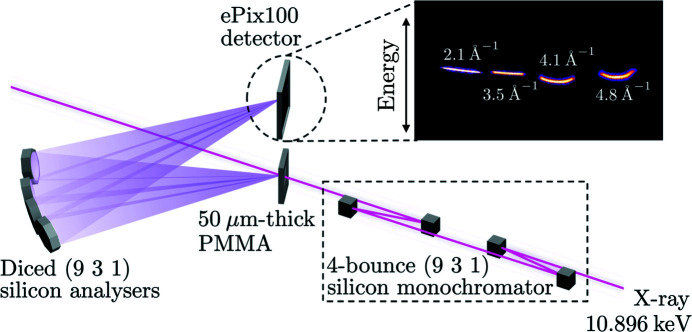
Schematic of the experimental setup used to perform milli-electronvolt inelastic X-ray scattering at the MEC endstation at the LCLS. X-ray pulses at 10.896 keV are first monochromated using a four-bounce (931) silicon monochromator arranged in a non-dispersive configuration and positioned at a glancing angle of incidence of 87.9°. Monochromatic X-ray pulses are then incident on a 50 µm-thick PMMA sample. Scattered photons are finally collected by four diced (931) silicon analysers and focused on an ePix100 detector (Carini *et al.*, 2016 ▸). The sample is oriented at a 22° angle such that the scattering vector **Q** is parallel to the [010] crystallographic direction at the 44° analyser location. The inset shows the raw data collected for each analyser. The curvature of the traces at *Q* = 4.1 Å^−1^ and *Q* = 4.8 Å^−1^ is postulated to arise from defocusing effects as these analysers could not be positioned at best focus.

The general layout of the experimental platform to perform milli-electronvolt (meV) inelastic X-ray scattering at XFELs is presented in Fig. 1 ▸ and described in detail by McBride *et al.* (2018 ▸) and Wollenweber *et al.* (2021 ▸). The tens of femto­seconds X-ray pulses at 10.896 keV produced by the LCLS are first incident on a four-bounce silicon single-crystal monochromator at a glancing angle of incidence of 87.9° to reduce the spectral bandwidth of the incident radiation down to the characteristic energy of inelastic modes (∼10 meV). These are phonons in solids (Baron, 2009 ▸), acoustic modes in liquids (Monaco *et al.*, 1999 ▸), or ion acoustic modes in dense plasmas (Gregori & Gericke, 2009 ▸). The monochromator is made of two (931) single-crystal silicon channel cuts arranged in a (+ − − +) non-dispersive configuration. For such experiments, four-bounce monochromators are preferably used as they do not introduce spatial offset of the X-ray beam.

X-ray pulses are then focused at the target plane location to a 20 µm spot using beryllium compound refractive lenses located 4 m upstream of the vacuum chamber with an estimated transmission of ∼30%. The X-ray focusing optics are described in detail by Heimann *et al.* (2016 ▸). For this experiment, the monochromator had to be positioned downstream of the X-ray focusing optics, resulting in a focusing beam, with a ∼200 µrad opening angle, to be incident on the first crystal. Given its non-dispersive configuration, the monochromator acts as both an energy filter and an angular filter, thus setting the angular divergence of the beam at the target plane to be ∼40 µrad and was estimated using the dynamical theory of X-ray diffraction. It should be noted that, ideally, one would position the monochromator upstream of the X-ray focusing optics as it would increase the number photons on target.

The samples were mounted on a six-axis hexapod which was manually tilted by 22° to align the [100] crystallographic direction of crystalline Au with the momentum transfer probed at 44° from the direct X-ray beam in the equatorial plane. Photons scattered from the samples are collected and energy-dispersed using spherically curved diced single-crystal (931) silicon analysers (ESRF Crystal Laboratory). They were operated at a glancing angle of incidence of 87.9°, *i.e.* close to backscattering, in a Johann geometry and placed in the 2 m-diameter vacuum chamber at the MEC endstation to probe specific momentum transfers, *Q*. From X-ray diffraction theory, photons with different energies will be reflected at different glancing angles of incidence, allowing one to measure the spectrum of the scattered light (Verbeni *et al.*, 2005 ▸). The analysers have a radius of curvature of 1 m and consist of thousands of flat single crystals of silicon 1.65 mm × 1.65 mm in size arranged on the surface of a sphere, thus creating a 10 cm-diameter spherically curved crystal. Using this geometry, the spectrum measured by each flat crystal is superimposed on the detector thus improving the recorded signal by the number of flat crystals. The analysers were mounted to provide pitch and yaw axes of rotation. These axes were used to position the focused image of the analysers on the X-ray detector.

In contrast to previous experiments which used the (533) reflection of silicon (McBride *et al.*, 2018 ▸; Wollenweber *et al.*, 2021 ▸), the (931) reflection of silicon was chosen for this experiment to balance the required energy resolution of the spectrometer and the number of photons recorded on the detector per X-ray pulse. It is the lowest reflection that could be used to resolve phonon modes in ambient Au, with a simulated energy resolution of 22 meV. One could obtain a higher energy resolution using this backscattering geometry by designing a spectrometer with a higher-order Bragg reflection combined with harder X-ray photons. However, this would be at the expense of the number of photons recorded on the detector, which we aim to optimize in this manuscript. For experiments attempting to combine the ultra-bright, ultra-short X-ray pulses delivered by an XFEL with a low-repetition-rate laser driver, such as those designed to investigate transient high-pressure, high-temperature states of matter, this reduction in photometrics is a serious limitation and makes the investigation of such systems extremely challenging. Indeed, the energy of the X-ray pulses delivered by the LCLS decreases above ∼10.6 keV (according to the LCLS estimated performance of the undulators), the quantum efficiency of the X-ray detector reaches its maximum at ∼10 keV (Blaj *et al.*, 2016 ▸), and the crystal reflectivity decreases at harder X-ray energies (Shvyd’ko, 2010 ▸).

Photons reflected by the analysers are finally focused onto a pixellated ePix100 detector with 50 µm × 50 µm pixels (Carini *et al.*, 2016 ▸). It should be noted that smaller pixels would decrease the energy increment for each pixel and thus increase the number of experimental data point recorded for each spectrum. For the measurements discussed here, the sample, the spherically curved diced analysers, and the detector are all placed on a 1 m Rowland circle. This choice was dictated by the diameter of the vacuum chamber available at the MEC endstation.

**Figure 2:**
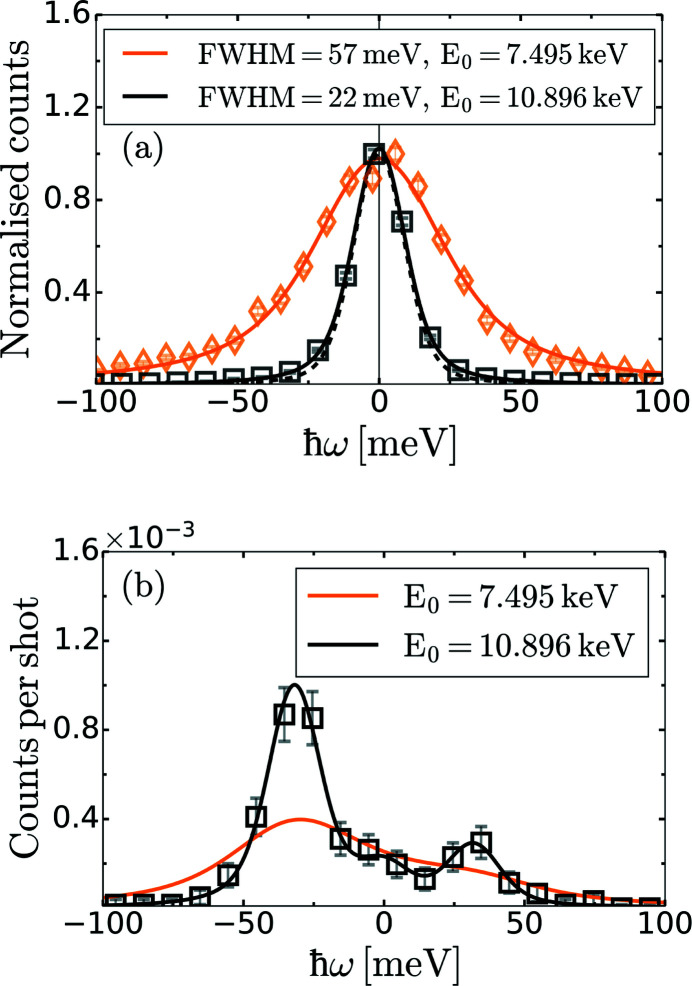
(*a*) Instrument functions measured on a 50 µm-thick PMMA sample at *Q* ≃ 2 Å^−1^ using the (533) reflection of silicon at 7.5 keV (orange diamonds) and the (931) reflection of silicon at 10.9 keV (black squares). The solid lines correspond to the best fit to the data assuming a pseudo-Voigt lineshape and the black dashed line shows the simulated instrument function. (*b*) Inelastic spectrum measured from a 10 µm-thick Fe sample at ambient conditions (dark squares) to highlight the effect of the improved energy resolution. The solid black line corresponds to the best fit to the data using the model described by Descamps *et al.* (2020 ▸). The orange line corresponds to the spectrum one would expect using the energy resolution demonstrated at 7.5 keV. In both figures, each symbol corresponds to one pixel on the X-ray detector.

The energy resolution expected for the spectrometer, including the performance of a matched monochromator, and taking into account the different contributions to the energy resolution, as detailed by Huotari *et al.* (2005 ▸), was simulated using an in-house ray-tracing algorithm which included treatment of the X-ray crystals within the frame of the dynamical theory of X-ray diffraction (Shvyd’ko, 2010 ▸; Authier, 2001 ▸). The experimentally measured instrument function is shown by the black squares in Fig. 2 ▸(*a*). Each square corresponds to one pixel on the detector. The best fit to the data assuming a pseudo-Voigt lineshape is shown with the solid black line, from which one finds the energy resolution, defined as the full width at half-maximum, to be 22 meV. This instrument function is found to be in good agreement with simulations shown by the solid black dashed line. The use of the (931) reflection of silicon leads to a 2.5-fold improvement of the energy resolution compared with the value obtained with the (533) reflection of silicon at 7.5 keV and shown in orange.

The different contributions to the energy resolution are summarized in Table 1 ▸. The incident bandwidth was calculated from the characteristics of the incident X-ray radiation and the reflectivity profile of the four-bounce monochromator in energy and acceptance angle space. For this calculation, an incident radiation at 10.895 keV with an energy bandwidth of 1.4 eV, *i.e.* in the hard X-ray self-seeding beam mode (see Section 3.1), and an opening angle of 200 µrad was used.

The shot-to-shot stability of the X-ray beam was monitored using the induced ultrafast ionization of a 100 µm YAG window located at the sample position. Upon irradiation by the X-ray pulse, the optical transmission of the window changes over the X-ray spot area, thus allowing one to measure the X-ray pulse position at the target plane on a shot-to-shot basis. It was found to be ∼5 µm in the vertical direction and ∼6 µm in the horizontal direction. It should be noted that these values are small enough to not contribute to the broadening of the instrument function reported in Table 1 ▸.

To highlight the improved instrument function, the inelastic scattering spectrum measured with a 10 µm-thick polycrystalline iron (Fe) sample at ambient conditions is shown Fig. 2 ▸(*b*). The black squares were collected at a momentum transfer of *Q* = 2.1 Å^−1^ and correspond to the accumulation of 4 × 10^4^ X-ray shots. The solid black line shows the best fit to the data using the model described by Descamps *et al.* (2020 ▸), from which one finds the phonon mode energy to be 32 ± 1 meV, consistent with phonon modes in b.c.c.-Fe (Minkiewicz *et al.*, 1967 ▸). Furthermore, using the principle of detailed balance, the temperature is measured to be 286 ± 30 K, in good agreement with ambient temperature. For comparison, the spectrum one would expect with the 57 meV energy resolution achieved previously (McBride *et al.*, 2018 ▸; Wollenweber *et al.*, 2021 ▸) is shown in orange. One can observe a broad inelastic feature from which the extraction of quantitative information can be challenging.

Iron was chosen here for its importance in planetary science as it is considered to be the main constituent of the Earth’s core (inner and outer) (Stevenson, 1981 ▸; Williams & Jeanloz, 1990 ▸). This measurement constitutes a preliminary step towards the investigation of Fe at extreme conditions. As an example, shock compressing Fe to 100 GPa on the Hugoniot would increase the lattice temperature to ∼1800 K and the bulk sound speed to ∼7 km s^−1^ (Brown & McQueen, 1986 ▸). Using the latter value, phonon modes in the first Brillouin zone at 32 meV are expected to increase in energy with pressure, reaching 51 meV at 100 GPa. Using these values, the ratio of the anti-Stokes intensity to the Stokes intensity, as given by the principle of detailed balance, is found to be ∼70%, and therefore measurable experimentally. It is finally mentioned that the temperature measurement using the principle of detailed balance might become important when investigating compressed systems for which the temperature remains low such as the ones generated using quasi-isentropic compression (Wang *et al.*, 2013 ▸) or double-shock compression (Kraus *et al.*, 2017 ▸).

## 3. Strategies to improve the photometrics

As mentioned in the previous section, the choice of the reflecting planes for the silicon optics cannot be arbitrary as one needs to balance the energy resolution of the spectrometer and the number of photons collected on the detector per X-ray pulse. Here, the choice was motivated by the highest energy resolution required to resolve phonon modes in ambient crystalline gold. In the following sections, strategies to maximize the number of photons recorded on the detector per X-ray shot without sacrificing the energy resolution are presented.

The number of photons recorded on the detector, *N*_Det_, for high-resolution inelastic X-ray scattering is given by (1) (Monaco *et al.*, 1999 ▸),

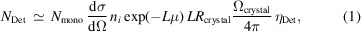

where *N*_mono_ is the number of photons after the monochromator, 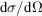 is the differential scattering cross section, *n*_*i*_ is the density of scatterers, *L* is the thickness of the target, μ is the attenuation coefficient at the corresponding X-ray energy, *R*_crystal_ is the reflectivity of the analyser, Ω_crystal_ is the solid angle covered by the analyser, and η_Det_ is the detector quantum efficiency. Out of all these parameters, *N*_mono_ and 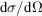 can be maximized by using the correct beam mode of operation of the XFEL and by a careful positioning of the analysers in the vacuum chamber. The other parameters are either set by the choice of the crystal reflection, the detector, or the characteristics of the sample which must often be matched to accommodate the laser drivers in extreme conditions experiments.

### 3.1. Selection of the XFEL beam mode

**Figure 3:**
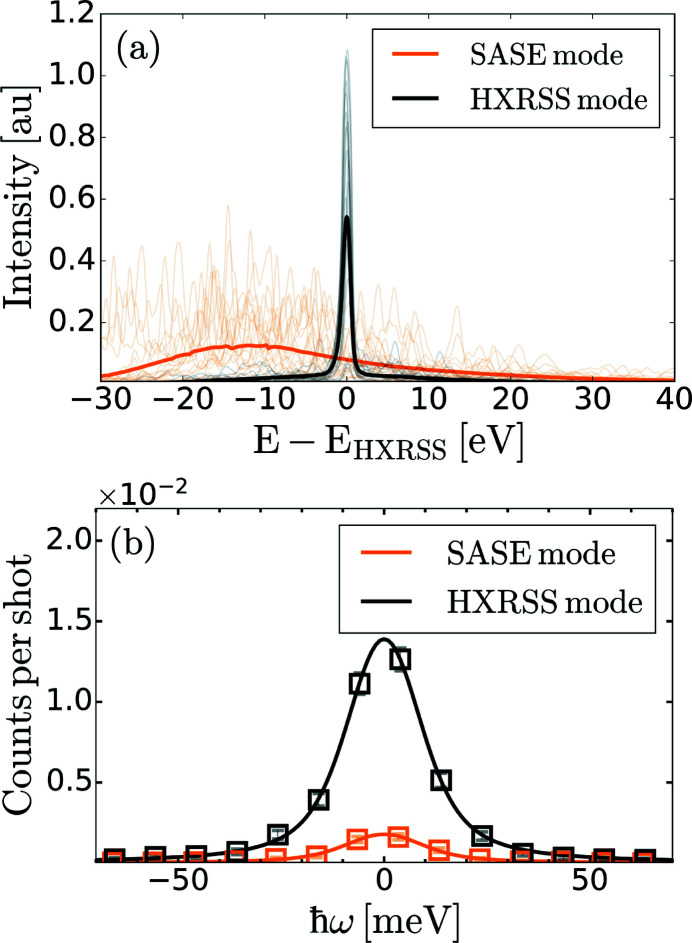
(*a*) Example spectra of the SASE beam mode (orange) and the HXRSS beam mode (black). The faint lines correspond to single-shot spectra recorded using a bent silicon spectrometer upstream of the MEC endstation at the LCLS (Zhu *et al.*, 2012 ▸). The thick lines correspond to the average spectra from 10^4^ shots. For clarity, only 20 single-shot spectra are shown. (*b*) Number of photons scattered from a 50 µm-thick PMMA sample at *Q* = 3.5 Å^−1^ using the SASE beam mode (orange squares) and using the HXRSS beam mode (black squares). The solid line corresponds to a fit to the experimental data using a pseudo-Voigt lineshape.

First, we will consider the number of photons after the monochromator. The monochromator is designed to reflect photons with energies within ∼8 meV of the incident energy, *E*_0_ = 10.896 keV. Its throughput depends on both the reflectivity of each of its crystals and the characteristics of the incident X-ray radiation. The latter can be maximized by using the appropriate beam mode of operation at XFELs. Here, we will focus the discussion on the two beam modes relevant for high-resolution inelastic X-ray scattering experiments: the self-amplified spontaneous emission (SASE) beam mode and the hard X-ray self-seeding (HXRSS) beam mode (Amann *et al.*, 2012 ▸). Compared with the SASE beam mode, the HXRSS beam mode provides X-ray pulses with a spectral bandwidth 20 to 30 times smaller than the characteristic bandwidth of the SASE beam (measured to be 1.3 eV for the HXRSS beam mode and 26 eV for the SASE beam mode in the spectra shown in Fig. 3 ▸). However, this reduces the number of photons per pulse to ∼30% (measured at the exit of the undulator to be 2.5 × 10^11^ photons per pulse for the HXRSS beam mode and 7.5 × 10^11^ photons per pulse for the SASE beam mode). It is important to note that the energy bandwidth of the monochromator is typically much smaller than the spectral bandwidth of the incident X-ray radiation, regardless of the beam mode of operation.

Typical examples of spectra in the SASE beam mode and the HXRSS beam mode are displayed in Fig. 3 ▸(*a*). It shows 20 single-shot measurements for each mode (faint lines) along with the average spectrum collected using 10^4^ X-ray pulses (thick solid lines). The spectra were measured using a curved silicon spectrometer positioned upstream of the MEC endstation (Zhu *et al.*, 2012 ▸). Comparing the two spectra, the average brightness is larger in the HXRSS beam mode but with larger shot-to-shot fluctuations in the X-ray photon energy, *E*_HXRSS_ (measured to be 0.4 eV). If *E*_HXRSS_ is offset from the resonant energy of the monochromator by more than 1.2 eV, then the thick black line would fall below the thick orange line in Fig. 3 ▸(*a*) and the throughput of the monochromator with the HXRSS mode would be smaller than the one obtained using the SASE beam mode. In the case of the SASE beam mode, this problem does not exist as the spectral bandwidth of the incoming radiation is much broader than typical X-ray energy fluctuations, *i.e.* the SASE bandwidth is 26 eV compared with fluctuations on the order of 0.4 eV.

Since high-resolution inelastic X-ray scattering measurements require the accumulation of many X-ray shots, the average number of photons recorded on the detector is the key quantity. In order to determine which beam mode is favorable, we measure the spectrum from a 50 µm-thick polymethyl methacrylate (PMMA) sample at *Q* = 3.5 Å^−1^ using both the SASE beam mode and the HXRSS beam mode. The results are shown in Fig. 3 ▸(*b*). After fine adjustment of the HXRSS energy to the resonance energy of the monochromator, the use of the HXRSS beam mode demonstrated a seven-fold improvement in the photometrics compared with the SASE beam mode.

### 3.2. Positioning of the analysers

**Figure 4:**
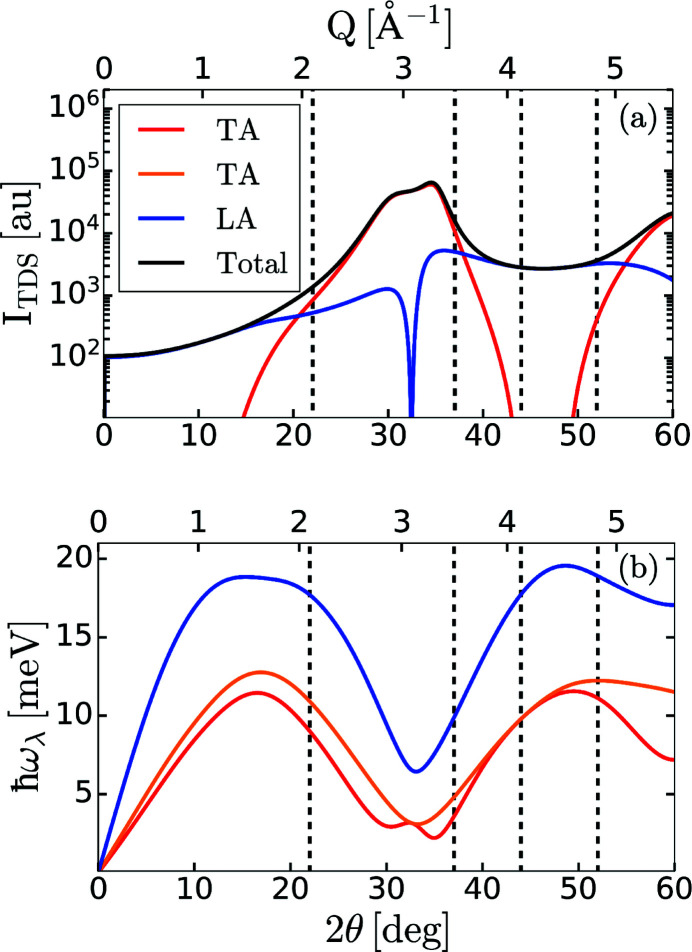
(*a*) Thermal diffuse scattering intensity calculated for gold, *I*_TDS_, as a function of the angular offset from the direct X-ray beam in the equatorial plane, 2θ. (*b*) Corresponding phonon dispersion relation. Blue corresponds to the longitudinal acoustic (LA) mode, orange and red correspond to the transverse acoustic (TA) modes. The solid black line in (*a*) corresponds to the summation over all the branches. The dashed vertical black lines in (*a*) and (*b*) represent the angular offset for the analysers used in the discussed experiment.

In the previous section, we have seen that the use of the HXRSS beam mode of operation available at XFELs provided an improvement of the photometrics. In this section, we will consider the effect of the positioning of the analysers on the number of photons recorded on the detector. It is important to note that the energy resolution is independent of the position of the analysers in the experimental chamber as long as they are at best focus. From equation (1), *N*_Det_ scales with the differential scattering cross section, 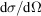 . This latter quantity depends, in a non-trivial way, on the probed scattering vector, **Q**, which in turns depends on the angles at which the analysers are placed inside the experimental chamber. The differential cross section is proportional to the X-ray scattering intensity which, in the case of crystalline samples, reduces, to first order, to the thermal diffuse scattering (TDS) intensity, *I*_TDS_, and is given by equation (2) (Warren *et al.*, 1967 ▸), 

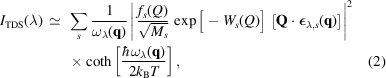

where *s* indicates the number of atoms in the primitive unit cell, λ identifies each phonon branch, and *Q* is the magnitude of the scattering vector. The quantities ω_λ_(**q**) and **ε**_λ,*s*_(**q**) correspond to the phonon frequency and phonon polarization vector at wavevector **q**, where **q** is expressed in the first Brillouin zone. Finally, *f*_*s*_(*Q*) is the X-ray atomic form factor at *Q*, *M*_*s*_ is the mass of atom *s*, and 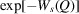 is the Debye–Waller factor. An example of the simulated thermal diffuse scattering intensity along with the corresponding phonon energies is shown in Fig. 4 ▸ for gold (Au), as it has been the subject of numerous theoretical (Recoules *et al.*, 2006 ▸; Smirnov, 2020 ▸) and experimental (Ernstorfer *et al.*, 2009 ▸; Mo *et al.*, 2021 ▸) studies at extreme conditions. The calculation was performed for a single-crystal Au sample with the [100] crystallographic direction oriented at 2θ = 22°, where θ is the angular offset from the direct X-ray beam in the equatorial plane of the experimental chamber. The phonon frequencies and phonon polarization vectors were computed by diagonalizing the dynamical matrix expressed within the Born Von Karman framework (Warren *et al.*, 1967 ▸; Baron, 2009 ▸, 2020 ▸) using the phonopy package (Togo & Tanaka, 2015 ▸). The dynamical matrices were constructed from interatomic force constants computed using density functional perturbation theory (DFPT) implemented in *QUANTUM ESPRESSO* (Giannozzi *et al.*, 2009 ▸, 2017 ▸). These calculations are based on a norm-conserving Vanderbilt pseudopotential for Au (Hamann, 2013 ▸) and a 7 × 7 × 7 Monkhorst–Pack **k**-point mesh. It is important to note that calculations of the thermal diffuse scattering rely on knowledge of the sample properties and can be challenging to obtain for systems under extreme conditions.

By inspecting the phonon energies in Fig. 4 ▸(*b*), one notices that the longitudinal acoustic mode (blue line) is the most energetic mode and, given the expected energy resolution of the spectrometer, the analysers should be positioned to be mostly sensitive to this mode. From Fig. 4 ▸(*a*), one finds two regions of 2θ where the total TDS intensity (black line) is dominated by the longitudinal acoustic (LA) mode: 0° ≤ 2θ ≤ 15° (0 Å^−1^ 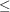 *Q* 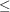 1.4 Å^−1^) and 40° ≤ 2θ ≤ 50° (3.8 Å 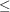 *Q* 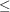 4.7 Å^−1^). Since the TDS intensity is larger in the second region, the analysers were preferentially positioned at 37° (*Q* = 3.5 Å^−1^), 44° (*Q* = 4.1 Å^−1^) and *Q* = 52° (*Q* = 4.8 Å^−1^) to accommodate for their physical size. A fourth analyser was placed at lower angle, 2θ = 22° (*Q* = 2.1 Å^−1^), due to limited space in the experimental chamber at higher angles.

**Figure 5:**
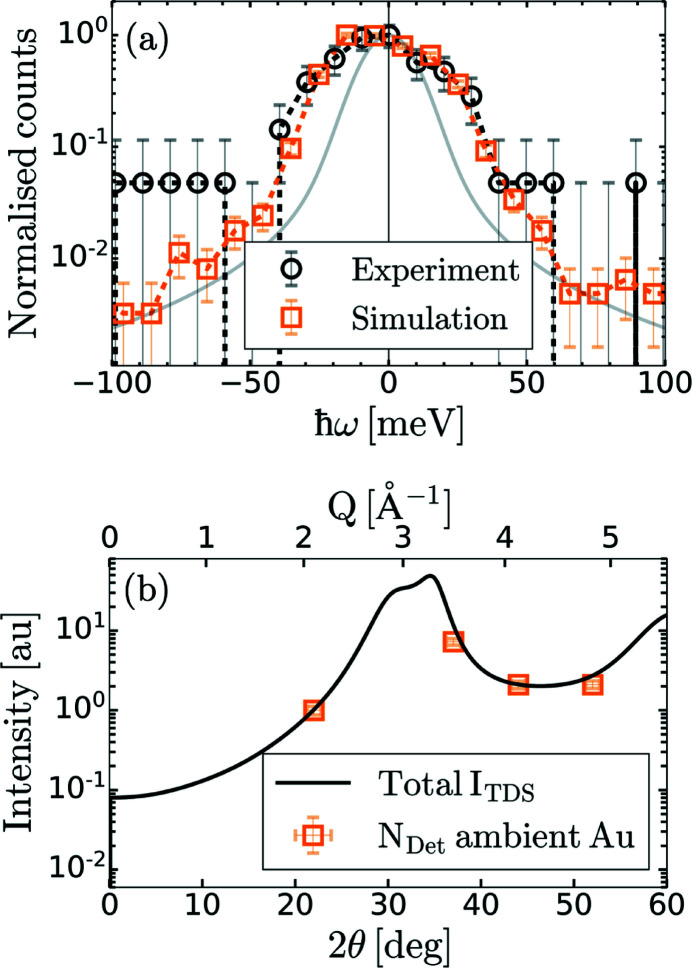
(*a*) Inelastic X-ray scattering spectrum recorded on a 50 nm-thick textured Au sample at 2.1 Å^−1^ and at ambient conditions (black) along with ray-tracing simulations (orange). The measured instrument function is shown in gray for comparison. (*b*) Calculated thermal diffuse scattering intensity, *I*_TDS_, for equation (2) normalized by the value at 2.1 Å^−1^ (22°) (black) along with the experimentally recorded number of photons on the detector, *N*_Det_, normalized by the value at 2.1 Å^−1^ (orange squares).

Applying the aforementioned considerations, the inelastic spectrum collected on a 50 nm-thick quasi-single-crystal Au sample at ambient conditions and at *Q* = 2.1 Å^−1^ (2θ = 22°) is shown with black open circles in Fig. 5 ▸(*a*). The experimental spectrum corresponds to the accumulation of 8.5 × 10^4^ X-ray shots collected at 120 Hz. The sample thickness was chosen to match the penetration depth of optical radiation at a wavelength of 400 nm in solid density gold. The samples were characterized using *ex situ* transmission electron microscopy measurements and revealed micrometre-size grains preferentially orientated along the [100] crystallographic direction. Given the dimensions of the X-ray spot at the target plane, only a few grains will be illuminated, thus explaining the quasi-single-crystal behavior of the samples. The experimental spectrum is compared with ray-tracing simulations on single-crystal Au (orange data set). For each ray and for each phonon branch, the phonon frequency and the TDS intensity are calculated using equation (2). The incident X-ray photon energy is then shifted by the phonon energy of one of the branches drawn at random based on their respective *I*_TDS_ values. The incident X-ray photon is then either blue-shifted or red-shifted to account for the asymmetry of the spectrum expected from the principle of detailed balance at room temperature. The experimental and simulated spectra are in good agreement, indicating that the broadening of the experimentally measured spectrum is consistent with phonon modes in ambient Au, and that the designed energy resolution is sufficient for such measurements.

In addition, we compare the number of photons accumulated on the detector for each analyser with the simulated TDS intensity as shown in Fig. 5 ▸(*b*). The good agreement between the experimental data and the simulations further support the quasi-single-crystal nature of the samples. The number of photons on the detector is at least two-fold larger at higher angles. It highlights the importance of a careful positioning of the analysers when maximizing the number of photons on the detector for low data acquisition rate experiments.

## 4. Conclusions

We presented a 2.5-fold improved energy resolution compared with previous experiments at similar XFEL instruments. We discussed aspects of the experimental design that can be adjusted to improve the number of recorded photons on the detector. This discussion was motivated by the smaller data acquisition rate expected when coupling such measurements with energetic driver lasers used to investigate matter in extreme conditions. The improvements in photometrics were demonstrated using experimental data collected on 50 nm-thick gold sample. By a careful selection of the energy resolution of the spectrometer and positioning of the analysers in the experimental chamber, one can increase the number of photons recorded on the detector. By further considering the self-seeding beam mode available at XFEL facilities, one can increase the photometrics by more than ten-fold. The improvements discussed in this manuscript will be vital when combining this technique with high-repetition-rate laser drivers available at the MEC endstation at LCLS (Glenzer *et al.*, 2016 ▸; Nagler *et al.*, 2015 ▸) and the HED instrument at European XFEL [10 Hz repetition rate short-pulse laser system, ReLaX (Laso Garcia *et al.*, 2021 ▸), and 10 Hz repetition rate long-pulse laser system, DiPOLE (Phillips *et al.*, 2019 ▸)], thus making high-resolution inelastic X-ray scattering experiments on systems at extreme conditions more feasible. Future improvements will come with the demonstration of an X-ray laser oscillator, which has long been postulated (Huang & Ruth, 2006 ▸). Such a scheme, if operated in a near backscattering geometry, would produce ∼1 mJ Fourier transform limited pulses with a tens to hundreds of meV energy bandwidth. Such improvements would remove the need for a monochromator and increase the number of photons recorded on the detector by several orders of magnitude. Such schemes currently under development include the XFELO project (Adams *et al.*, 2019 ▸) and the XLO project (Halavanau *et al.*, 2020 ▸).

## Figures and Tables

**Table 1:** Contributions to the energy resolution of the high-resolution spectrometer using a 50 µm-thick PMMA sample The different contributions are defined by Huotari *et al.* (2005 ▸) and Moretti Sala *et al.* (2018 ▸).

Contribution	Δ*E* (meV)
Analyser Darwin width	14
Source size	8
Pixel size	10
Johann aberration	5
Spectrometer	20
Incident bandwidth	11
Total	22
